# Investigation of a measles outbreak in a highly vaccinated middle school, France, 2023

**DOI:** 10.2807/1560-7917.ES.2025.30.46.2500130

**Published:** 2025-11-20

**Authors:** Thomas Bénet, Erica Fougère, Magali Gounon, Alexandra Thabuis, Christelle Vauloup-Fellous, Laura Zanetti, Isabelle Parent du Chatelet, Nathalie Ragozin, Julia Dina, Christine Saura

**Affiliations:** 1 Santé publique France, Cellule Auvergne-Rhône-Alpes, Lyon, France; 2 Agence régionale de Santé Auvergne-Rhône-Alpes, Lyon, France; 3 Division of Virology, WHO Rubella National Reference Laboratory, Paris Saclay University Hospital, APHP, Villejuif, France; 4 Paris Saclay University, INSERM U1184, CEA, Center for Immunology of Viral, Auto-immune, Hematological and Bacterial diseases (IMVA-HB/IDMIT), Fontenay-aux-Roses, France; 5 Santé publique France, Département des maladies infectieuses, Saint-Maurice, France; 6 Department of Virology, Normandie University UNICAEN, CHU de CAEN Normandie, INSERM UMR 1311, DYNAMICURE, Caen, France; * These authors contributed equally to this work and share first authorship.

**Keywords:** measles, outbreak, France, secondary school, investigation, MMR vaccine, vaccine effectiveness

## Abstract

In September 2023, a measles outbreak occurred in a middle school (Rhône valley, France), with the index case returning from Asia. Investigations involved case validation, virological analyses, contact tracing and checking vaccination records to determine measles vaccination coverage (VC) and attack rates (AR). Among 643 students, 49 measles cases occurred between 6 September and 18 October 2023 (AR = 7.6%). Two-dose vaccination coverage was 93.5% (601/643). Virological analyses confirmed the measles strains’ clonality (genotype D8) and the imported origin. Concordance between health record vaccination status and immunological profile was established for 27 cases. In a sub-cohort of children (all cases and 309 non-cases), AR was 100% in unvaccinated children, and 43.7%, 16.5% and 3.2% among two-dose vaccinated children with the first dose administered at 6–8, 9–11 and ≥ 12 months, respectively. After multivariate binomial regression, vaccine effectiveness (VE) was 96.4% (95% confidence interval (CI): 91.4–98.5) after two-dose vaccination with the first dose at ≥ 12 months, confirming long-term effectiveness of measles-mumps-rubella vaccines. When the first dose was given at 9–11 and 6–8 months, respectively, VE was 83.3% (95% CI: 74.3–89.2) and 60.7% (95% CI: 10.6–82.7). This measles epidemic mainly affected unvaccinated or two-dose vaccinated children with first dose administered before age 12 months.

**Key public health message**

**What did you want to address in this study and why?**

We wanted to describe a large measles outbreak during a period marked by low measles circulation in France in 2023. This study aimed to emphasize the preventive impact of measles-mumps-rubella (MMR) vaccination and to investigate the effect of a child’s age at their first measles-containing vaccine (MCV) dose on their long-term protection against measles during an outbreak.

**What have we learnt from this study?**

This measles epidemic was unusual because half of the 64 cases occurred among middle school students vaccinated with two MCV doses. The protection (vaccine effectiveness) was lower among students who had received the first MCV dose at an age younger than 12 months, while it was higher among those vaccinated later.

**What are the implications of your findings for public health?**

This study provides additional evidence that there is a risk of measles re-emergence if the first MCV dose is given too early (i.e. before 12 months of age) and that catch-up vaccination is needed in such cases, particularly in countries where early vaccination was historically recommended in specific circumstances. Future evaluations should estimate the proportion of children/teenagers with a lower level of protection against measles, by region or country.

## Introduction

Measles is a highly contagious viral infection caused by a paramyxovirus, with a basic reproductive rate of 12–18 [1]. Transmission occurs mainly through the air or by direct contact with the nasopharyngeal secretions of infected people. The virus remains active in the air and on contaminated surfaces for up to 2 h. Because the disease is highly contagious, outbreaks are common. After a most common incubation period of 10–14 days (range: 7–23 days), nonspecific signs such as fever, cough and conjunctivitis usually appear, followed by a maculopapular rash. The contagious phase begins on the day before onset and continues up to 5 days after the start of the rash. Measles complications are quite common and serious, and can include pneumonia and acute encephalitis [2], with an estimation of 107,500 deaths worldwide in 2023 [3]. In addition, up to 90% of susceptible individuals (unvaccinated or without past measles) exposed to a case may become infected [4]. Since 2005, as part of the World Health Organization (WHO) measles elimination programme, the French National Health Agency (Santé publique France) has been conducting measles surveillance, in the framework of the WHO’s measles elimination programme, relying on the mandatory reporting of infectious diseases. In November 2024, the WHO expressed concern about the rapid spread of measles with an estimated number of 10.3 million cases in 2023 [5] and local epidemics in Europe.

In France, a major epidemic occurred from 2008 to 2011 [6], severely impacting southern France and particularly the Auvergne-Rhône-Alpes region [7]. In 2018 and 2019, measles cases increased again [8], followed by an interruption of virus circulation from 2020 to 2022 during the COVID-19 pandemic. From the start of the 2023 school year, France experienced a marked increase in measles cases, resulting in a total number of reported cases in 2023 that was eightfold higher than in 2022. Among them, 26% cases were imported, with transmission chains of varying length that remained limited (range: 2–64 cases) [9]. 

Two-dose measles containing vaccine (MCV) is mandatory in France, at age 12 months and 16–18 months. However, during the measles epidemic in 2010 and 2011, it was temporarily recommended to administer the first MCV dose at age 9 months for children in collective care facilities. In the Auvergne-Rhône-Alpes region, two-dose vaccination coverage against measles at age 33 months was 89% in 2023 (82–91% depending on the department), it was 85.7% in France [10], below the 95% WHO objective for measles elimination [11]. In 2023, the measles elimination status was considered as ‘interrupted transmission’ in France [12].

The primary objective of this study was to describe the measles outbreak occurring between September and November 2023 in a middle school in the Auvergne-Rhône-Alpes Region, France, where most children were vaccinated against measles. Secondary objectives were (i) to estimate vaccine effectiveness (VE) by age at first dose, (ii) to describe the infection control measures implemented locally, and (iii) to determine the biological validity of the vaccination status recorded in the vaccination records.

## Outbreak detection

On 18 September 2023, the Auvergne-Rhône-Alpes Regional Health Agency (Agence Régionale de Santé, ARS) was informed about a laboratory-confirmed measles case in a vaccinated (two doses of MCV) middle school student. The investigation rapidly revealed that their first MCV dose was given early, at 8 months of age. This notification was made as part of the national measles surveillance. Each suspected or confirmed measles case must be declared by health professionals in order to implement infection control measures and for surveillance purposes; this system is described elsewhere [11,13]. An investigation with contact tracing and control measures was initiated, but other cases were reported to the ARS in the following days.

## Methods

### Epidemiological investigations and data collection

The case definition was based on the notifiable disease reporting criteria [14]. According to the national definition, a case of clinical measles was defined as a person with a fever ≥ 38.5 °C, maculopapular rash and at least one of the following signs: conjunctivitis, coryza, cough and Koplik’s sign [10]. Laboratory-confirmed measles was defined as a person presenting with clinical measles, confirmed by at least one of the following: specific anti-measles IgM antibodies in saliva or blood, seroconversion, minimum fourfold increase in serum IgG titres, or a positive measles RT-PCR in saliva, blood or nasopharyngeal swab. An epidemiologically confirmed case was a clinical case who had been in contact with a laboratory-confirmed measles case in the 7–18 days before the onset of rash. 

Inclusion criteria for epidemiological investigation were: (i) case of measles meeting the definition of a laboratory- or epidemiologically confirmed case and declared from 29 August 2023 onwards; (ii) contact with the imported index case or with the middle school (students, school staff and outside contacts). The Auvergne-Rhône-Alpes ARS and the Regional Office of Santé publique conducted the investigations and control measures. The following data were collected for each measles case: date of onset, clinical signs, complications, hospitalisation, links between cases, types of sample (saliva, serum or nasopharyngeal swabs), biological results of the laboratory investigation (measles RNA detection, serological testing), vaccination status (number of doses, vaccination dates, vaccine brand, batch number, doctor) and history of measles.

On-site data collection from the middle school students’ health records was performed on 28 September 2023. Epidemiologists verified the information on vaccination status and history of measles by consulting the children’s health records booklets (616 of 643 (96%) had their booklets, 22 (3%) had no vaccine booklet, and five (1%) were not present the day of data collection). If the vaccine booklet was not available, we researched other information sources (middle school health registry or asking the parents). We created an individual database of all middle school students and collected the following data: sex, age, school level (from Year 7 (students aged 11–12 years) to Year 10 (students aged 14–15 years)), class, number of MCV doses, vaccination dates and history of measles. 

For a sub-cohort of middle school students (i.e. almost all children in Years 7 and 8 and a random sample in Years 9 and 10 (sample fraction: 50%)), we collected additional data for each administered vaccine dose from health and vaccine records: vaccine brand name, vaccination date, batch number, expiry date, name and municipality of the vaccinating doctor. This sub-cohort is referred to as a ‘sub-cohort with complete vaccination data’. Time constraints prevented us from collecting all vaccine traceability data for all middle school students, as the health records were only available for a single school day.

### Laboratory investigations

As part of this outbreak, all samples from the investigated cases taken in laboratories were sent to the measles–mumps–rubella (MMR) national reference centre in Caen (France) to confirm the diagnosis, to perform genotyping based on N-450 sequence and molecular epidemiological analyses, and to characterise the immune response against measles (anti-measles IgM and IgG titres, IgG avidity tests), as well as anti-mumps and anti-rubella IgG titres. The avidity test permitted us to distinguish between primary infection (positive IgG with low avidity) and vaccine failure (IgG positive with high avidity). For each notified case, we made efforts to obtain samples so that detection and molecular characterisation of the measles virus (MeV) and the serological response could be carried out.

### Statistical analysis

Categorical variables were expressed as numbers and compared using chi-square or Fischer’s exact test. Continuous variables were described as mean (+/− standard deviation (SD)) and compared using t-test. We performed descriptive analysis and calculated stratified attack rates (AR = n measles cases/n students %). To estimate VE, was performed an additional analysis in the sub-cohort with complete vaccination data for each vaccine dose: type, delay between doses, and prescriber (detailed for doctors who administered MMR vaccines in at least five children, otherwise grouped as ‘others’). Middle school students from the sub-cohort were compared with the other students to identify possible selection bias. The analysis from the sub-cohort excluded healthy children with a history of measles in childhood and those with incomplete vaccination data (lost health records, n = 3). We modelled the risk of measles in this sub-cohort according to vaccination status (unvaccinated, one vaccine dose, two vaccine doses) and with additional stratification for the age at first MCV dose (6–8, 9–11 and ≥ 12 months). Uni- and multivariate analyses were performed by negative binomial regression with the 95% confidence interval estimated by the bootstrap method (50 replications). The explained variable was the presence/absence of measles, while the explanatory variable was vaccination profile. Multivariate models were adjusted for sex, school year and age to account for possible confusion and the differential sample fraction according to school level. Two-by-two interactions were tested. From these risks, was estimated VE = (1 − adjusted incidence rate ratio) × 100. All tests were two-sided, p < 0.05 was considered significant. Analyses were performed in Stata 18.0 (StataCorp LP).

## Results

### Outbreak description

**Figure 1:**
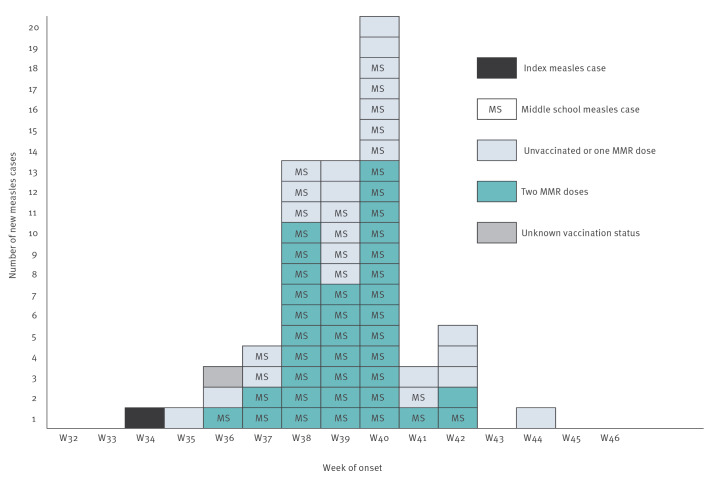
Weekly epidemic curve of measles cases by date of onset, Region Auvergne-Rhône-Alpes, France, 2023 (n = 64)

Overall, 64 measles cases were identified (57 children, seven adults), with 57 laboratory-confirmed cases (47 by RT-PCR MeV detection and 10 by detection of MeV-specific IgM antibodies, mean delay between onset and sample collection: 2 days) and seven cases with an epidemiological link to a laboratory-confirmed case. Among them 50 occurred in the college (49 middle school students and one supervising adult) and 14 in other settings. Cases were aged 7–52 years (IQR: 11–14), 35 were male and 29 were female (male:female ratio: 1.2). The onset date extended from 26 August (week 34) to 2 November 2023 (week 44) (Figure 1), with the end of the outbreak declared on 8 December 2023.

MMR: measles-mumps-rubella vaccine.

**Figure 2:**
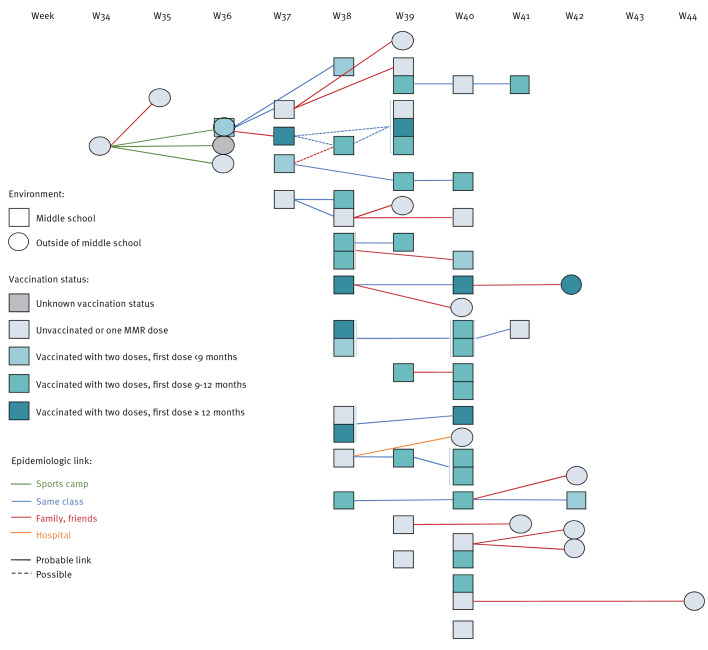
Chains of transmission of measles cases per week of outbreak, Region Auvergne-Rhône-Alpes, France, 2023 (n = 64)

The infection in the index case (around 10-years-old, unvaccinated laboratory-confirmed measles case) was an imported infection acquired in Asia, and all other cases were infected in France. The first secondary cases included the index case’s sibling, one middle school student and two other individuals. Among these four secondary cases, only the index case’s sibling had participated in the same sporting event in late August. Another of the four participants in the same sporting event as the index case in late August, despite being vaccinated, introduced the virus to the middle school students, resulting in 49 further cases within the school community and 10 further cases among students’ family members and friends. The reconstructed chains of transmission are depicted in Figure 2.

MMR: measles-mumps-rubella vaccine.

For 40 confirmed cases, we identified a variant of MeV genotype D8 (MVs/Oita.JPN/30.22 exact match) that had been circulating in Indonesia since 2022 (GenBank accession number: LC830470), confirming the clonality and imported origin. A phylogenetic tree of the measles outbreak is appended in Supplementary Figure S1. 

There was no notable difference in disease severity between vaccinated and unvaccinated cases; for further details regarding the frequency of clinical signs reported by measles cases and their severity refer to Supplementary Table S1. Three unvaccinated cases were hospitalised (two middle school students and one adult), including one who developed pneumonitis as a measles-related complication, with a favourable clinical outcome. No deaths were reported. The vaccination status of the 64 cases, reported in the health record, was as follows: 37 vaccinated with two doses, 24 unvaccinated, none vaccinated with one dose and three with unknown vaccine status.

A high concordance between the status reported in the MMR vaccine status on health records and the serological results was found among 27 of 29 measles cases for whom these were available and complete serological data were obtained. For the immune status of measles cases according to the national reference centre’s analyses and concordance with vaccination status according to the health record for college cases and cases outside the middle school, we refer to Supplementary Table S2. Six of seven non-vaccinated cases were negative for anti-mumps IgG, and one of the seven was negative for anti-rubella IgG antibodies. Among 20 vaccinated cases with one or two MCV doses, seven were positive for anti-mumps IgG and one was positive for anti-rubella IgG antibodies. Among the 18 cases vaccinated with two doses, two were negative and 16 were positive for anti-measles IgG. Among those with positive anti-measles IgG, 14 presented with high avidity index (> 70%) and two with low avidity index (< 30%). Thus, among the vaccinated cases with complete serological results, four could be considered as primary vaccine failure (two IgG-negative and two IgG-positive with low avidity) and 14 as secondary vaccine failure (IgG-positive with high avidity).

### Analysis of the measles outbreak in the middle school population 

The mean age of the 643 middle school students was 12.7 years, and the male:female ratio was 1.2. Among them, 601 (93.5%) were vaccinated against measles with two doses, seven (1.1%) with one dose, 14 (2.2%) were not vaccinated and 21 (3.3%) had unknown status. Two middle school students had a documented history of measles in childhood; both were vaccinated with two MCV doses (first dose at 6 months of age for one, unknown age for the second) and did not report measles during this outbreak.

Fifty measles cases were reported in the school, with onset between 6 September and 18 October 2023: 49 students and a supervising adult. The vaccination status of the 49 student cases was as follows: 36 were vaccinated with two doses and 13 not vaccinated. None had received a third MCV dose, and the supervising adult was not vaccinated.

The overall AR in the middle school was 7.6% (calculated based on the 49 students), with no significant difference related to sex, age or school year (Table 1). The measles AR differed depending on the number of MCV doses (p < 0.001), with an AR of 100% among unvaccinated students, 0% among those vaccinated with one dose and 5.8% among those vaccinated with two doses. The measles outbreak affected almost all middle school classes (0–5 cases per class, with the AR ranging from 0% to 17%. We append a detailed measles attack rate by school year in the middle school in Supplementary Figure S2. Two of 14 unvaccinated schoolchildren were hospitalised, whereas none of the 35 children who had received two doses of the vaccine were hospitalised.

**Table 1:** Number of patients and attack rates stratified according to the main characteristics of the middle school students, Region Auvergne-Rhône-Alpes, France, 2023 (n = 643)

Characteristics	Total number of middle school children	Number of measles cases	Attack rate (%)	p value^a^
Total	643	49	7.6	
Sex				
Female	294	19	6.5	0.31
Male	349	30	8.6	
Age				
< 11 years	63	5	7.9	0.92
11 years	150	10	6.7	
12 years	150	14	9.3	
13 years	165	12	7.3	
14–15 years	115	8	7.0	
School year				
Year 7	173	11	6.4	0.58
Year 8	152	13	8.6	
Year 9	143	14	9.8	
Year 10	175	11	6.3	
MMR vaccination status				
Unvaccinated	14	14	100	< 0.001
One vaccine dose	7	0	0	
Two vaccine doses	601	35	5.8	
Unknown	21	0	0	

MMR: measles-mumps-rubella vaccine.

^a^ No measles vs measles, after chi-square test. If there was no missing value, the missing value category is not shown.

### Vaccine effectiveness in middle school students with complete vaccination data

Among middle school students without measles, the sub-cohort of students with complete vaccination data (n = 309) did not differ from those with incomplete vaccination data (n = 285) with respect to sex distribution (p = 0.19), although their mean age was 0.9 years higher. Table 2 compares the characteristics between cases and non-cases in this sub-cohort. Measles cases did not differ from non-cases regarding sex, age, type of vaccine administered as the first/second dose, and delay between first and second dose. Average age at first dose was 10.8 months (standard deviation (SD): +/− 4.9) in cases and 14.4 months (SD: +/− 12.0) in vaccinated non-cases (p = 0.08). The average age at the second dose was 20.4 months (SD: +/− 9.7) in cases and 26.3 months (SD: +/− 18.1) in vaccinated non-cases (p = 0.06). Among vaccinated middle school students, no vaccinating doctor or vaccine batch was significantly associated with the risk of measles (data not shown), thus ruling out the hypothesis of health record falsification or batch issues.

**Table 2:** Comparison of measles cases vs non-cases among middle school students with complete vaccination data, Region Auvergne-Rhône-Alpes, France, 2023 (n = 358)

Characteristics	No measles		Measles		p value
	n	%	n	%	
Total	309	86.3	49	13.7	
Sex					
Female	151	48.9	19	38.8	0.19
Male	158	51.1	30	61.2	
Age					
< 11 years	21	6.8	5	10.2	0.10
11 years	44	14.2	10	20.4	
12 years	56	18.1	14	28.6	
13 years	91	29.4	12	24.5	
14–15 years	97	31.4	8	16.3	
MMR vaccination status					
Unvaccinated	0	0.0	14	28.6	< 0.001
One vaccine dose	7	2.3	0	0	
Two vaccine doses	302	97.7	35	71.4	
Age at first dose					
6–8 months	9	2.9	7	14.3	< 0.001
9–11 months	111	35.9	22	44.9	
≥ 12 months	182	58.9	6	12.2	
Missing values	7	2.3	14	28.6	
MCV brand, first dose^a^					
MMR-VAXPRO	110	35.6	13	37.1	0.83
PRIORIX	180	58.2	22	62.9	
MMR, unspecified	3	1.0	0	0	
Missing values	16	5.2	0	0	
MCV brand, second dose^a^					
MMR-VAXPRO	95	30.7	10	28.6	0.65
PRIORIX	188	60.8	25	71.4	
MMR, unspecified	4	1.3	0	0	
Missing values	22	7.3	0	0	
Delay between first and second MCV doses in months (SD)	12.9 (15.1)		9.6 (8.1)		0.20

MCV: measles containing vaccine; MMR: measles-mumps-rubella.

^a^ Comparisons and tests on vaccinated people with known information on the name of the vaccine. If there was no missing value, the missing value category was not shown.

The proportion of measles cases differed depending on MMR vaccination status in the sub-cohort (p < 0.001). Among children vaccinated with two doses at known dates, 4.7% (16/337) had received their first dose at age 6–8 months, 39.5% (133/337) at 9–11 months and 55.8% (188/337) at ≥ 12 months. Among children vaccinated with two doses, 82.9% (29/35) of cases were vaccinated before 12 months vs 39.7% (120/302) of non-cases (p < 0.001). In addition, the risk of measles was higher among children who had received their first MCV dose at 9 months (16 cases/60 vaccinated children; 27%), compared with those who had their first dose at 10 months (4/29; 14%) or 11 months (2/28; 7%; not significant, p = 0.25). Age at first dose was linked to the year of birth, as 108 of 159 (67.9%) children born in 2008 and 2009 received their first dose at ≥ 12 months, 21 of the 63 born in 2010 (during epidemic peak in 2011), 30 of the 63 of the 63 born in 2011 and 29 of the 53 born in 2012 and 2013 (p < 0.001).

After multivariate analysis (Table 3), MMR vaccination was significantly associated with a reduced risk of measles, independently of sex, age and school year, with a two-dose VE of 94.2% (95% CI: 92.2–95.7). The level of protection increased with increasing age at the first dose. During this outbreak, VE was estimated at > 95% (VE = 96.4%; 95% CI: 91.4–98.5) for a complete vaccination schedule with a first dose at ≥ 12 months, regardless of sex, current age and school year. The VE was lower for a first dose at 9–11 months (VE = 83.3%; 95% CI: 74.3–89.2) or 6–8 months (VE = 60.7%; 95%: 10.6–82.7).

**Table 3:** Vaccination protection according to age at first MCV dose for all middle school students (n = 643) and a sub-cohort with complete vaccination data (n = 358), Region Auvergne-Rhône-Alpes, France, 2023

Characteristics	Measles cases			Crude IRR (95% CI)	p value	Adjusted IRR^a^ (95% CI)	p value*	Estimated VE^b^ (95% CI)
	n	Total	%					
All middle school students (n = 643)								
**MMR vaccination status**								
Unvaccinated	14	14	100	1.0 (reference)				
One vaccine dose	0	7	0	NE				
Two vaccine doses	35	601	5.8	0.06 (0.04–0.08)	< 0.001	0.05 (0.03–0.09)	< 0.001	94.2% (92.2–95.7)
Unknown	0	21	0	NE				
Sub-cohort of middle school students with complete vaccination data (n = 358)								
**MMR vaccination status**								
Unvaccinated	14	14	100	1.0 (reference)				
One vaccine dose	0	7	0	NE				
**Two MCV doses**								
First dose at 6–8 months	7	16	43.8	0.44 (0.23–0.83)	0.01	0.39 (0.17–0.89)	0.026	60.7% (10.6–82.7)
First dose at 9–11 months	22	133	16.5	0.17 (0.11–0.23)	< 0.001	0.17 (0.11–0.26)	< 0.001	83.3% (74.3–89.2)
First dose at ≥ 12 months	6	188	3.2	0.04 (0.01–0.10)	< 0.001	0.04 (0.02–0.09)	< 0.001	96.4% (91.4–98.5)

CI: confidence interval; IRR: incidence rate ratio; MMR: measles-mumps-rubella; NE: not estimable; VE: vaccine effectiveness.

^a^ Models adjusted for sex, school year (7th, 8th, 9th, 10th) and age after multivariate negative binomial regression and estimation of 95% CI by bootstrap (50 replications). Reference: unvaccinated.

^b^ According to risk estimation in the multivariate model (VE = 1 − adjusted IRR) in the sub-cohort of 358 children.

## Outbreak control measures

All cases were investigated according to national recommendations for contact tracing, at-risk unvaccinated contacts received post-exposure prophylaxis (MMR vaccination), and no immunoglobulin was administrated [15]. On 17 October 2023, a decision support meeting was organised with local and national representatives and clinical experts who gave two recommendations to the ARS: (i) administering a third MCV dose to people born after 1980 with no history of measles who had received a first dose before age 12 months in departments affected by the outbreak, and (ii) implementing catch-up vaccination in all people born after 1980 who had not received two MCV doses. The population was informed about the recommendations by SMS or by health and scholar professionals. All healthcare professionals were informed about the opportunistic catch-up. A vaccination campaign was organised on 23 and 25 October 2023 at the hospital closest to the middle school as well as in the surrounding area: 31 people were vaccinated, including 17 children. According to reimbursement data for MCV from the National Health Data System (système national des données de santé) MMR vaccinations reimbursed in the department where the outbreak occurred almost doubled during the last quarter of 2023 compared with the same period in 2022. For a map of sectors analysed for monitoring MMR vaccinations and the number of monthly MMR vaccinations among 10–15-year-olds by area, we refer to Supplementary Figures S3-S4.

## Discussion

This study described the measles outbreak that occurred between September and November 2023 in a middle school where most children (93.5%) were vaccinated against MeV. This outbreak was the largest in the area since the major epidemic from 2008 to 2011 in the Auvergne-Rhône-Alpes region. Initial investigations identified the rapid spread of measles in a middle school where two-dose MMR vaccination coverage was particularly high, while viral spread was low in the community. The detection of the same MeV sequence variant of genotype D8 in 40 analysed middle school cases confirmed clonality. It should be noted that with high coverage, the overall number of cases drops notably, but a greater percentage of those cases will have been vaccinated, even though there is only a small fraction of vaccinated people who become cases. Seeing a growing proportion of vaccinated individuals among measles cases does not by itself mean that the vaccine is failing. Instead, it reflects how well the vaccine protects most people and how large the vaccinated group has become in a population with high coverage, as previously demonstrated [16].

The index case, returning from a leisure trip to Asia, was declared late, which delayed identification of the first-generation transmission. In the middle school, the first two generations of cases developed before the implementation of control measures, leading to rapid diffusion. Viral spread in the middle school was also facilitated by the confined classrooms and multiple contacts between students. In addition, a large proportion of vaccinated students received their first MCV dose before 12 months of age (149/358). As some children had measles despite vaccination it was difficult to convince the parents of unvaccinated children to follow the vaccine recommendations. In early October, given the large proportion of cases vaccinated with two doses, we investigated hypotheses such as falsification of health records or quality issues with some vaccine batches. However, these were rapidly ruled out after checking the health/vaccine booklets.

During this outbreak, the proportion of secondary vaccine failure was high [17,18]. We therefore hypothesised that the drop in long-term immune protection was linked to the administration of the first dose before 12 months of age, particularly among children born between 2008 and 2013, when the department was affected by a major measles epidemic (2008–2011). During this period in France, it was recommended to administer MCV from age 9 months for children in collective care facilities or from age 6 months as post-exposure prophylaxis. We should also mention that recent similar outbreaks in highly vaccinated populations are rarely reported from European countries [19,20], as most measles outbreaks are associated with sub-optimal MCV vaccination coverage or special populations [21-24]. However, these findings may be useful in France and in Europe as a remind of the importance of a catch-up programme for children who had received a first dose before the age of 12 months.

In the present outbreak, the long-term VE of MCV, more than 10 years after the first dose, was confirmed, as VE exceeded 95% (VE = 96.4%) for a complete vaccination schedule with the first dose at age ≥ 12 months. However, VE dropped to 83.3% when the first dose was given at the age of 9–11 months and to 60.7% for age 6–8 months. These results are consistent with several studies showing the long-term effectiveness of MMR vaccination and higher vaccine response rates with increasing age at first vaccination. In France, an evaluation conducted between 2017 and 2019 indicated that the long-term effectiveness of MMR vaccination remains high over time, being 95% among 2–5-year-olds and 91.4% among 26–31-year-olds [25]. The VE exceeded 99% up to 4 years after vaccination with two MCV doses (i.e. for children aged 6 years and vaccinated at around 2-years-old) and then decreased slowly to reach 97.8% 11–12 years after vaccination (i.e. for children aged 13–14 years) [25]. In a meta-analysis published in 2020, two-dose VE increased by 1.4% (95% CI: 0.0–2.7) for every month increase in age at the first MCV dose [26]. Another meta-analysis of epidemiological studies suggested lower VE in children vaccinated with two doses when the first dose was administered before age 12 months, 75% and 89% (versus 100% at ≥ 12 months) in two cohort studies [27]. In another study, the risk of measles was estimated to be three times higher (risk ratio = 3.48; 95% CI: 1.4–8.4) in the event of vaccination between 9 and 12 months compared with 12–14 months. In contrast, Carazo et al.’s meta-analysis of serological studies confirmed that the seroconversion rate is high regardless of age at administration of first MCV dose [27]. A Dutch study showed that in children vaccinated before age 12 months, the decline in neutralising antibodies was much faster and the avidity of IgG antibodies lower than in those vaccinated after age 12 months [28]. In countries with low levels of measles transmission, the WHO recommends that vaccination should be delayed until age 12–15 months to take advantage of the higher seroconversion rates achieved at this age [29]. In line with these recommendations, the current French vaccination schedule advocates MMR vaccination from the age of 12 months (except as post-exposure prophylaxis or for travellers): specifically, first MCV dose at 12 months and second dose between 16 and 18 months (minimum interval of 1 month between doses). Our results support the recommendation introduced in the French vaccination schedule in April 2024 that all individuals who received a first dose before 12 months complete their MMR vaccination to receive three dose of trivalent vaccine in total. In our study, none of the 149 children vaccinated before 12 months of age had received a third dose during childhood, highlighting a potential gap in health care professionals' knowledge to vaccination recommendations. Regarding severity, the hospitalisation rate was 5%. This rate is quite low, but the age category of most cases (10–14 years) is usually associated with lower hospitalisation rates compared with younger children (e.g. in the United States in 2025, 7% of measles cases aged between 5 and 19 years were hospitalised [30]). Among cases from the middle school, there was also a trend for higher hospitalisation rates among non-vaccinated compared with vaccinated children, which is in agreement with the literature [31].

The main strength of this study is its in-depth epidemiological investigation, including prospective data collection with the direct observation of vaccine and medical records, the confirmation of cases by the National Reference Centre, and estimations of VE adjusted on several determinants. Some limitations must be underlined. Firstly, the incomplete collection of precise data for the entire middle school population may have led to selection bias. However, comparative analysis revealed that the sub-cohort of middle school students with complete vaccination data did not differ from the other students, with the analyses adjusted to the school year. Secondly, this was a single centre outbreak, thus limiting generalisation to other settings. Thirdly, study power was limited for some analyses of VE (i.e. by month of first dose). Finally, the appearance of measles-specific IgG at the time of diagnosis, in the absence of previous serum samples, did not allow us to determine immune status before the outbreak. The MMR vaccination status, as recorded in health records, was cross-checked by detecting IgG antibodies specific to mumps and rubella, which serve as serological markers of an immune response following MMR vaccination. The presence of anti-mumps and anti-rubella antibodies was interpreted as evidence of MMR vaccination before the measles outbreak. However, this did not allow conclusions regarding the effectiveness of the vaccination for which the number of doses and dates of administration were recorded in the vaccination booklet.

## Conclusion

This measles outbreak immediately emerged as unusual due to the high number of cases among middle school students vaccinated with two MCV doses, while diffusion outside this establishment remained limited. Our results confirm the long-term effectiveness of MCV among middle school students who received a first MCV dose after 12 months of age, supporting MMR vaccination strategies in the context of international resurgence of measles. Our study also revealed significantly lower protection when the vaccine was administrated before 12 months of age. This outbreak underscores the need to implement catch-up vaccination programmes to remind healthcare professionals of the importance to better protect all individuals who received their first dose before 12 months of age—particularly in countries like France, where early vaccination was historically recommended in specific circumstances. Such programmes are essential to guarantee sustained long-term protection against measles and prevent future outbreaks.

## Data Availability

Epidemiological data are available upon reasonable request. Reads were deposited in the Genbank database under the study accession numbers PX480021–PX480061. The data for MVs/Sampang.IDN/41.22/9 are not publicly available.

## Supplementary Data

647000 Supplement
