# Supplementary material for: Investigation of a measles outbreak in a highly vaccinated middle school, France, 2023
Source: Euro Surveill. 2025 Nov 20;30(46):2500130. doi: 10.2807/1560-7917.ES.2025.30.46.2500130 (PMC12639275; doi:10.2807/1560-7917.ES.2025.30.46.2500130)
Supplement: Supplement [file 25-00130_FOUGERE_supplement.pdf]

Supplementary materials

Title: Investigation of a measles outbreak in a highly vaccinated middle school, France, 2023

This supplementary material is hosted by *Eurosurveillance* as supporting information alongside the article « Investigation of a measles outbreak in a highly vaccinated middle school, France, 2023 », on behalf of the authors, who remain responsible for the accuracy and appropriateness of the content. The same standards for ethics, copyright, attributions and permissions as for the article apply. Supplements are not edited by *Eurosurveillance* and the journal is not responsible for the maintenance of any links or email addresses provided therein.

Template

Supplementary Table S1. Frequency of clinical signs reported by measles cases and severity, Region Auvergne-Rhône-Alpes (France), 2023 (n=64)

Supplementary Table S2. Immune status of measles cases according to CNR MMR analyzes and concordance with vaccination status according to the health record for college cases and cases outside the middle school, Region Auvergne-Rhône-Alpes (France), 2023 (n=29)

Supplementary Table S3. Number of monthly MMR vaccinations among 10-15 year olds, in Drôme, Ardèche and the EPCI concerned in October and November 2023

Supplementary Figure S1. Phylogenetic tree, measles outbreak, Region Auvergne-Rhône-Alpes (France), 2023

Supplementary Figure S2. Measles attack rate by grade class (n=643), middle school, measles outbreak, Region Auvergne-Rhône-Alpes (France), 2023

Supplementary Figure S3. Map of sectors analysed for monitoring MMR vaccinations, departments affected by the outbreak (France), 2023

Supplementary Figure S4. Number of monthly MMR vaccinations among 10-15 year olds, in Ardèche (A), Drôme (B), and the administrative area (EPCI) concerned (C and D) in October and November 2023

Classified as ECDC NORMAL

Supplementary Table S1. Frequency of clinical signs reported by measles cases and severity, Region Auvergne-Rhône-Alpes (France), 2023 (n=64)

**Caracteristics, N (%)**	**Children (n=57)**				**Adults (n=7)**			
	**Vaccined (n=40)**		**Non vaccined (n=17)**		**Vaccinated (n=0)**		**Non vaccined (n=7)**	
	n	%	n	%	n	%	n	%
Eruption	39	98	16	94	-	-	7	100
Fever ≥ 38,5°C	34	85	17	100	-	-	7	100
Occulo-respiratory catarrh (cough, rhinitis, conjunctivitis)	31	78	15	88	-	-	4	57%
Hospitalisation	0	0	1	6	-	-	0	0
Pneumonia	0	0	1	6	-	-	1	14

Supplementary Table S2. Immune status of measles cases according to MMR NRC analyses and concordance with vaccination status according to the health record for college cases and cases outside the middle school, Region Auvergne-Rhône-Alpes (France), 2023 (n=29)

**Vaccinal status based on vaccine record**	**Number of measles cases**	**Number of serums analysed among cases**	**Number of cases non-immunised (NI) and previously immunised (PI) agains measles, according to NRC results**
Middle school			
Non vaccinated	13	5	5 NI
Vaccinated with MMR, 1 dose	0	-	-
Vaccinated with MMR, 2 doses	36	18	17 IA, 1NI
Unknwon vaccine status	1	1	1 NI
Total	50	24	
Other settings			
Non vaccinated	8	3	3 NI
Vaccinated with MMR, 1 dose	3	2	2 IA
Vaccinated with MMR, 2 doses	1	0	6
Unknwon vaccine status	2	0	2 IND
Total	14	5	
Overall			
Non vaccinated	21	8	8 NI
Vaccinated 1D	3	2	2 IA
Vaccinated 2D	37	18	17 IA, 1 NI
Vaccinated IND	3	1	1 NI
Total	64	29	

Supplementary Table S3. Number of monthly MMR vaccinations among 10-15 year olds, in Drôme, Ardèche and the EPCI concerned in October and November 2023

**Number of reimbursed MMR vaccines, 10-15 years**	**Monthly average. jan-21 to sept-23**	**Oct-23**	**Nov-23**	**Variation oct-nov-23***
Auvergne-Rhône-Alpes	1 038	1 067	965	
Ardèche	11	49	35	*+ 62*
Drôme	19	64	48	*+ 74*

* compared to expected (monthly average Jan-21_Sept-23)

Classified as ECDC NORMAL

Supplementary Figure S1. Phylogenetic tree, measles outbreak, Region Auvergne-Rhône-Alpes (France), 2023

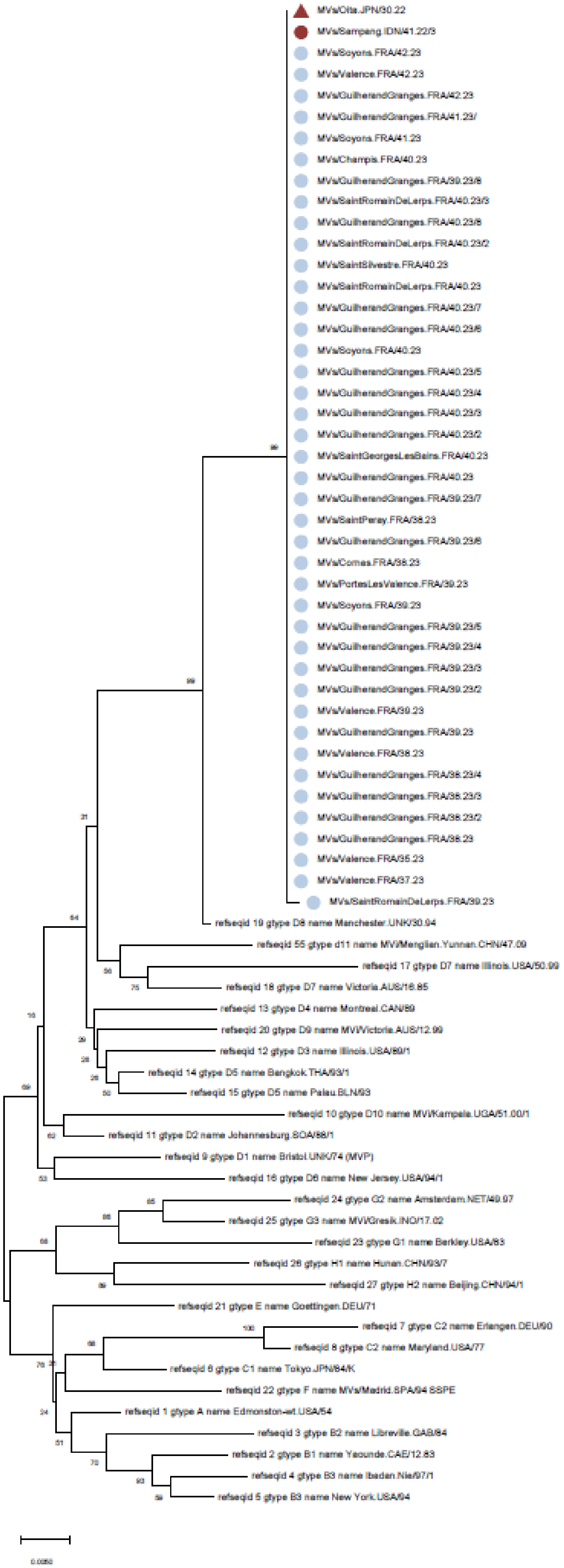

Classified as ECDC NORMAL

Supplementary Figure S2. Measles attack rate by grade class (n=643), middle school, measles outbreak, Region Auvergne-Rhône-Alpes (France), 2023

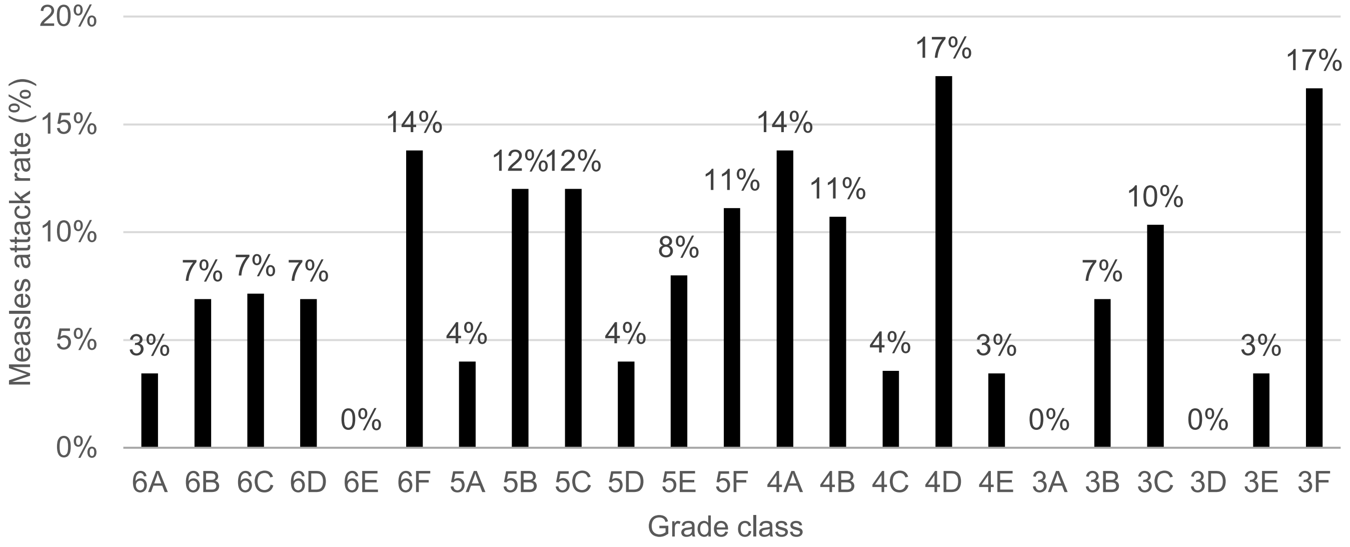

Supplementary Figure S3. Map of sectors analysed for monitoring MMR vaccinations, departments affected by the outbreak (France), 2023

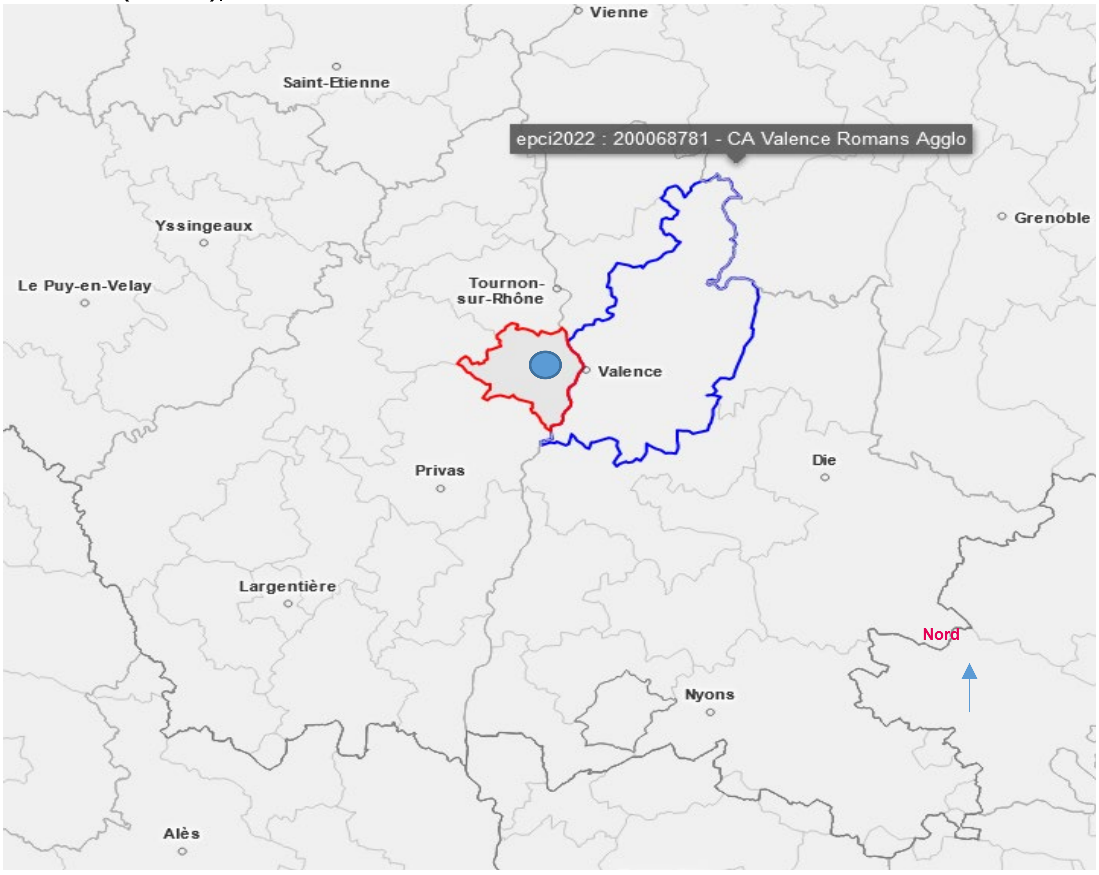

Red : administrative area, EPCI CC Rhône-Crusol.

Blue administrative area, EPCI CA Valence Romans Agglo

Classified as ECDC NORMAL

Supplementary Figure S4. Number of monthly MMR vaccinations among 10-15 year olds, in Ardèche (A), Drôme (B), and the administrative area (EPCI) concerned (C and D) in October and November 2023

A. Ardèche

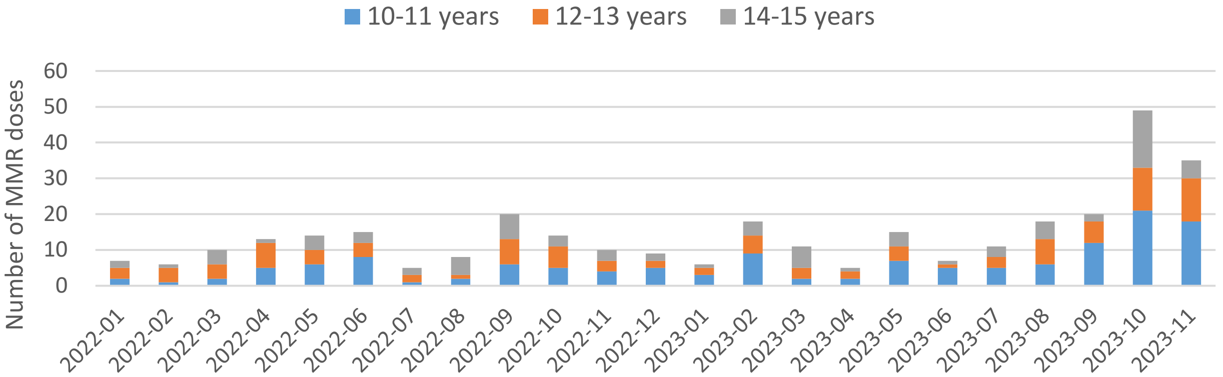

B. Drôme

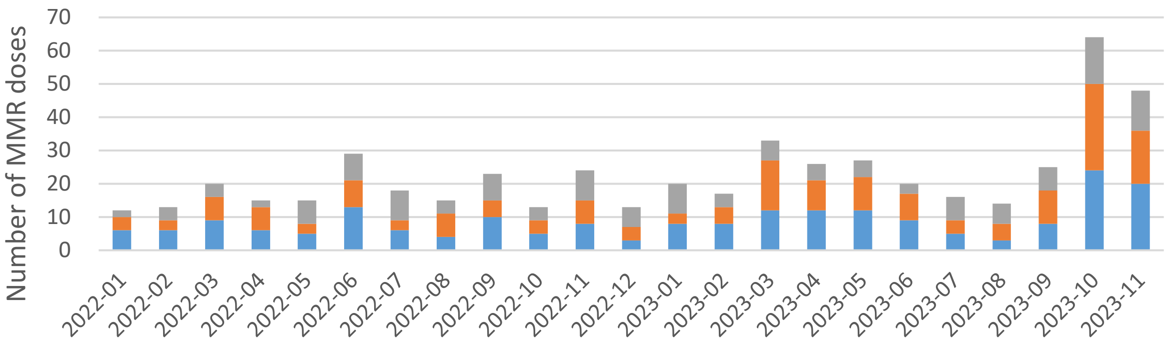

C. Administrative area EPCI CC Rhône-Crusol

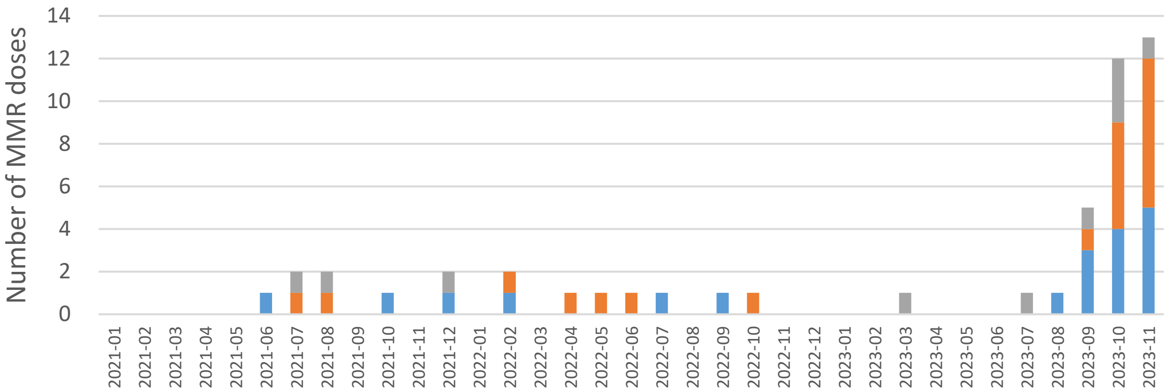

D. Administrative area EPCI CA Valence Romans Agglo

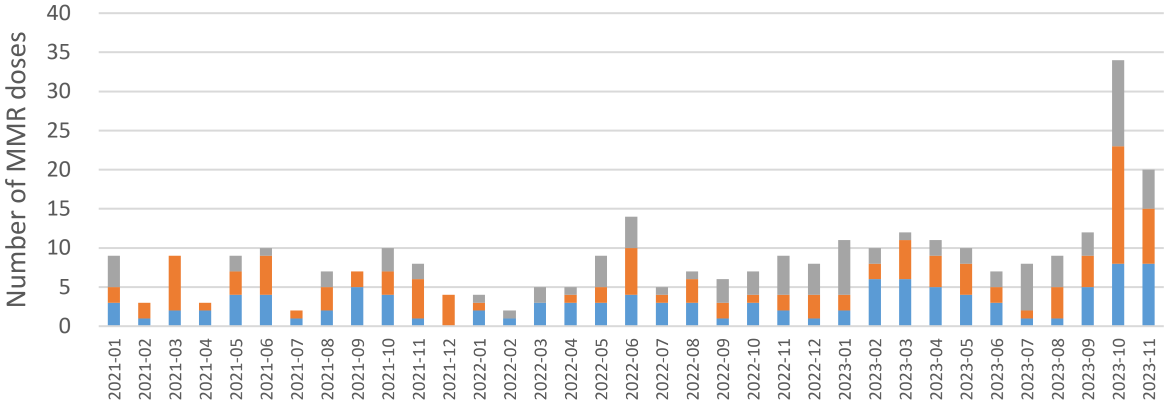

Classified as ECDC NORMAL
